# Highly Functionalized Modified Metal Oxides Polymeric Sensors for Potentiometric Determination of Letrozole in Commercial Oral Tablets and Biosamples

**DOI:** 10.3390/polym13091384

**Published:** 2021-04-23

**Authors:** Ahmed Mahmoud Shawky, Maha Farouk El-Tohamy

**Affiliations:** 1 Science and Technology Unit (STU), Umm Al-Qura University, Makkah 21955, Saudi Arabia; amesmail@uqu.edu.sa; 2 Department of Chemistry, College of Science, King Saud University, P.O. Box 22452, Riyadh 11495, Saudi Arabia

**Keywords:** letrozole, electrochemical analysis, metal oxide nanoparticles, potentiometric sensors, pharmaceutical formulations, biosamples

## Abstract

The advanced and high-functional activities of magnesium oxide and copper oxide nanoparticles encourage the extensive use of these metal oxides as remarkable electroactive materials in electrochemical and sensing detections. The current study described a comparative sensing activity and selectivity of modified coated wire membrane sensors enriched with magnesium oxide and copper oxide nanoparticles for quantifying the breast cancer medication letrozole (LTZ) in its pharmaceutical form and human plasma. The fabricated sensors were based on the incorporation of LTZ with phosphomolybdic acid (PMA) to form the electroactive complex letrozole-phosphomolybate (LTZ-PM) in the presence of *o*-nitrophenyloctyl ether (*o*-NPOE) as a solvent mediator. Under optimum conditions, the modified sensors LTZ-PM-MgONPs and LTZ-PM-CuONPs demonstrated linear relationships of 1.0 × 10^−8^–1.0 × 10^−2^ and 1.0 × 10^−10^–1.0 × 10^−2^ mol L^−1^, respectively. Least square equations were calculated as E_mV_ = (56.4 ± 0.7) log [LTZ] + 569.6 and E_mV_ = (58.7 ± 0.3) log [LTZ] + 692.6 for LTZ-PM-MgONPs and LTZ-PM-CuONPs, respectively. The conventional type LTZ-PM showed a potential response E_mV_ = (53.3 ± 0.5) log [LTZ] + 451.4 over concentration range of 1.0 × 10^−6^–1.0 × 10^−2^ mol L^−1^. The suggested sensors were successfully used to determine LTZ in pharmaceutical formulations and biosamples. Method validation ensured the suitability of the suggested potentiometric sensors.

## 1. Introduction

Nano-scale materials are considered a potential key in sensors, material construction, electronics, drug delivery systems, and cancer diagnosis. The nano size material exhibits different and amazing properties that are considered a possible solution for many current problems and can be an essential contribution to solve some global and environmental challenges. These unique properties can potentially modify our life cycle and can be utilized for the construction of ultra-sensitive sensors [1,2,3,4].

Magnesium oxide (MgO) is a basic oxide that has various applications. It has a promising potential as a caustic adsorbent of toxic chemical wastes. Due to the characteristic structures and versatile properties of MgO nanoparticles, they displayed exceptional optical, electronic, magnetic, thermal, mechanical and chemical features. Therefore, MgO nanoparticles have significantly been utilized in catalysis, toxic wastes remediation and refractory material industries [5,6,7].

Copper oxide (CuO) is a strong p-type semiconductor. It has rewarded substantial attention due to its outstanding optical, electrical, physical, and magnetic properties. Thus, it is heavily utilized in different purposes such as catalysis [8], conversions of solar energy [9], sensors [10] and emissions [11]. Nevertheless, these characteristics can be enhanced by synthesizing CuO in nanoparticles for better performance when compared with their bulk counterparts. Different approaches have been proposed to fabricate nanoparticles in several sizes and shapes such as thermal oxidation [12], sonochemical [13], combustion [14] and quick precipitation [15]. The multi-functional physicochemical features, large surface area, powerful binding properties and high isoelectric stability of magnesium oxide nanoparticles (MgONPs) and copper oxide nanoparticles (CuONPs) enhanced their usage in various analytical probes including electrochemical sensors [16,17], biomedical applications [18,19] and drug delivery systems [20,21].

The typical potentiometric electrodes for detecting medicinal drugs, organic or inorganic compounds are the classical form of self-powered electrodes that do not need any external energy sources for their work [22]. The potentiometric measurements of these electrodes are achieved as a result of analyte accumulation under the effect of an electrostatic mechanism causing the production of potential difference between working electrode surface and the surface of reference electrode [22]. The diverse structure and size of the synthesized MgONPs and CuONPs provide an advanced competence for the construction of various catalytic sensing systems. These systems exhibit rapid, ultrasensitive and selective characteristics such as low detection limits, wide concentration ranges, higher recoveries percentage, strong reproducibility, and functionality under room temperature conditions [23,24].

Letrozole (LTZ) is an oral non-steroidal medication for breast cancer therapy after surgery. It acts as aromatase inhibitor and prevents estrogen production. The action of this medication is very specific and does not inhibit the formation of corticosteroids [25]. LTZ was previously quantified using various analytical techniques, including chromatographic separation such as high-performance thin layer chromatography [26], reversed phase liquid chromatography [27,28], liquid chromatography coupled with tandem mass spectrometry [29,30], and capillary zone electrophoresis [31]. Furthermore, different spectrophotometric methods were reported for the determination of LTZ in various media [32,33,34,35,36]. However, very few articles were concerned with potentiometric determination of LTZ [37]. These techniques exhibited several analytical advantages such as excellent sensitivity to quantify organic and inorganic substances with useful and wide linear concentration ranges. However, they still possess certain drawbacks such as the need for high technical skills and long analytical time as well as the consumption of large solvent quantities.

Currently, determination and quantification of dosage form medications using modified metal oxide sensing electrodes have gained major attention. The objective of this study is to fabricate highly sensitive and selective modified coated wire sensors enriched with MgONPs and CuONPs. Those sensors were applied for electrochemical quantification of the breast cancer medication LTZ in its tablets and biosamples. Moreover, method validation was carried out to evaluate the validity of the suggested modified sensors. Additionally, a comparative study was carried out between the enriched sensors with metal oxide nanoparticles and the conventional fabricated ones.

## 2. Experimental

### 2.1. Chemicals

The purest form of breast cancer medication LTZ and its Femara^®^ tablets (2.5 mg Letrozole/tablet) were obtained from Saudi Pharmaceutical Distribution Co. Ltd. (Novartise, Jeddah, Saudi Arabia). Magnesium sulfate, sodium hydroxide and copper nitrate (Cu(NO_3_)_2_·3H_2_O) were supplied by BDH (Poole, UK). Various solvents and chemicals including methanol 99.9%, acetone 99.9%, ethanol 99.9%, tetrahydrofuran (THF) 97.0%, ortho-nitrophenyloctyl ether (*o*-NPOE), hydrochloric acid 37%, phosphomolybdic acid as well as high molecular weight polyvinyl chloride (PVC) were supplied by Sigma Aldrich (Hamburg, Germany). The blood samples were collected from patients in King Khalid hospitals (Riyadh, Saudi Arabia), and the research ethics committee at King Saud University, KSA (KSU-REC-002-E, 2020) approved the study.

### 2.2. Instruments

A digital pH meter HANNA model 211 (HANNA instruments, Smithfield, RI, USA) was used to perform all the potentiometric measurements. Metrohm pH-meter model 744 (Metrohm Co., Herisau, Switzerland) was used to control the pH conditions of the analyte samples. The electrochemical systems were comprised of a fabricated indicator electrode in conjunction with a silver/silver chloride (Ag/AgCl) as a reference electrode. Spectrophotometer (Shimadzu Corporation, Kyoto, Japan), Spectrum BX spectrometer, (PerkinElmer, Waltham, WA, USA), Transmission electron microscope (TEM) (JEM-2100F, JEOL Ltd., Akishima, Tokyo, Japan), Scanning Electron Microscope (SEM) (JSM-7610F; JEOL, Tokyo, Japan), and X-ray diffraction (XRD) (Shimadzu XRD-6000 diffractometer, Kyoto, Japan) were used for nanoparticles characterization. Energy-Dispersive X-Ray Spectroscopy (EDX) analysis was obtained by a SEM microscope (JSM-7610F; JEOL, Tokyo, Japan) connected with EDX to ensure the presence of magnesium and copper in samples.

### 2.3. Synthesis of Magnesium Oxide and Copper Oxide Nanoparticles

Magnesium hydroxide was prepared by mixing 50 mL of each 5% magnesium sulfate (MgSO_4_) and 5% sodium hydroxide (NaOH) solutions under magnetic stirring for 3 h. The formed magnesium hydroxide was heated in a hot air oven for another 3 h at 100 °C. This precursor was calcined in a muffle furnace at 500 °C to obtain MgONPs [38].

The synthesis of CuONPs using copper nitrate was conducted by preparing 100 mL of 1.0 × 10^−1^ mol L^−1^ of copper nitrate in deionized water. A solution of 1.0 × 10^−1^ mol L^−1^ sodium hydroxide was added dropwise with continuous stirring. The black precipitate was observed when the pH elevated to 14. Deionized water and absolute ethanol were used to wash and neutralize the formed precipitate, and then dried at 80 °C for 16 h [39].

### 2.4. Characterization of Nanoparticles

Spectrophotometric detection at a wavelength range of 200–500 nm using a UV 2450 Spectrophotometer (Shimadzu Corporation, Kyoto, Japan) was carried out to ensure the formation of MgONPs and CuONPs. Fourier-Transform Infrared spectroscopy (FT-IR) spectra was used to determine the predicted functional groups that appear in the −prepared MgONPs and CuONPs. Microscopic examination under TEM and SEM was performed to study the surface structure, shape and particle size of both MgONPs and CuONPs.

### 2.5. Preparation of Standard Drug Solution

A standard 1.0 × 10^−2^ mol L^−1^ of LTZ solution (pH = 4) was obtained by dissolving 0.285 g of LTZ in 100 mL acidic distilled water (HCL:water, 1:3 *v*/*v*). Analytical solutions in the ranges of 1.0 × 10^−6^–1.0 × 10^−2^, 1.0 × 10^−8^–1.0 × 10^−2^ and 1.0 × 10^−10^–1.0 × 10^−2^ mol L^−1^ were prepared by performing serial dilutions using the same solvent. The experimental studies were carried out using a conventional LTZ-PM, modified LTZ-PM-MgONPs and LTZ-PM-CuONPs coated wire sensors, respectively.

### 2.6. Preparation of Electroactive Complex

The electroactive complex (ion pair) of LTZ-PM was prepared by mixing equal volumes (50 mL) of 1.0 × 10^−2^ mol L^−1^ of LTZ acidic solution (pH = 4) with 1.0 × 10^−2^ mol L^−1^ of PMA solution. A yellowish precipitate of LTZ-PM complex was obtained. The resulted precipitate was filtrated using Schleicher and Schuelfilter paper No. 595 Ø150 mm, washed three times with distilled water, and air dried at ambient temperature overnight.

### 2.7. Membrane Composition and Sensor Fabrication

**Figure 1:**
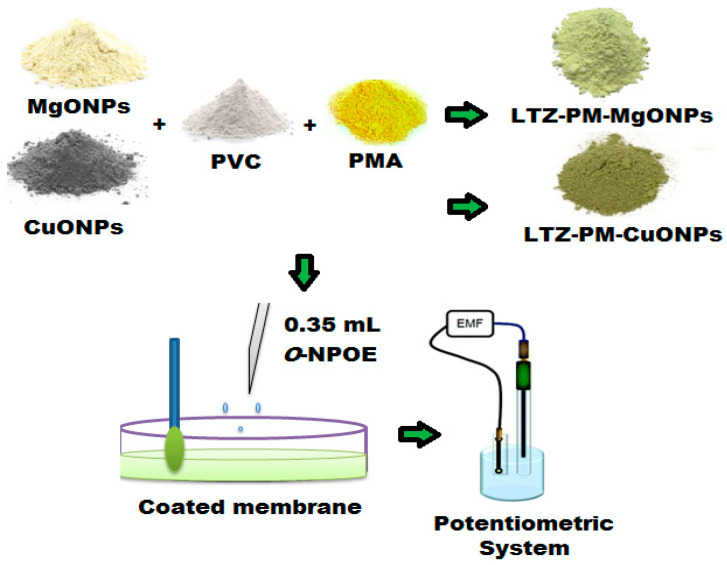
Illustrated the construction of the modified sensor and its potentiometric system.

Three different coated wire sensors based on LTZ-PM, LTZ-PM-MgONPs and LTZ-PM-CuONPs were fabricated by mixing 190 mg of high molecular weight polyvinyl chloride (PVC), 10 mg of ion-pair (LTZ-PM), and 0.35 mL of plasticizer *o*-NPOE in 5 mL of THF. The mixed solution was purred in a Petri dish (3 cm in diameter) and allowed to evaporate until the formation of an oily membrane solution. An Al wire was polished and acetone cleaned, then dipped several times in the membrane mixture to construct the conventional LTZ-PM sensor. To fabricate the modified sensors, a plastic membrane mixture containing MgONPs or CuONPs (5 g), PVC (190 mg), LTZ-PM-MgONPs or LTZ-PM-CuONPs ion-pair (10 mg) and *o*-NPOE plasticizer (0.35 mL) in 5 mL of THF were prepared. A well homogeneous dispersed membrane mixture was obtained by continuously stirring it for 15 min at room temperature. The formed polymeric membrane mixtures were used to formulate a thin layer on the surface of the sensors. After drying, sensors were immersed in the coated membrane mixture (several times) to form a thick coated wire membrane. The fabricated sensors were designed as follows: Al wire/modified coated membrane/test solution//Ag/AgCl reference electrode (Figure 1).

### 2.8. Calibration Graphs

Twenty-five mL of 1.0 × 10^−10^–1.0 × 10^−2^ mol L^−1^ LTZ standard solution was analyzed using the fabricated sensors separately in conjunction with Ag/AgCl as a reference electrode. The calibration graphs of each sensor were plotted (Microsoft office Excel 2010) using the potential readings as a function of –logarithm LTZ concentrations.

### 2.9. Optimization of Potential Readings Condition

To evaluate the pH effect, 0.1 mol L^−1^ hydrochloric acid was used to acidify 1.0 × 10^−4^ mol L^−1^ of LTZ test solution. The potential readings were recorded after elevating the pH using 0.1 mol L^−1^ of sodium hydroxide and the fabricated sensors were separately used in conjunction with Ag/AgCl reference electrode and combined glass electrode for measuring pH values. pH graphs were plotted (Microsoft office Excel 2010) using the pH values as a function of potential readings of each sensor.

The separate solution method [40] was followed to evaluate the selectivity of the suggested sensors. The tolerable values of various interfering species including cations, sugars, amino acids and co-formulated compounds were calculated using the following equation:

Log K^pot^ = (E_2_ − E_1_)/S + Log [LTZ] − Log [B^z+^]^1/z^ (1)

The equation represented as selectivity coefficient (K^pot^), potential reading of 1.0 × 10^−3^ mol L^−1^ LTZ (E_1_), potential reading of 1.0 × 10^−3^ mol L^−1^ of interfering species (E_2_), interfering ions (B^z+^) and slope of the calibration graph (S).

The dynamic response time was investigated by measuring the potential response of the tested drug using a concentration range of 1.0 × 10^−10^–1.0 × 10^−2^ mol L^−1^.

### 2.10. Analysis of LTZ in Femara^®^ Tablets

Twenty Femara^®^ tablets (2.5 mg/tablet) were milled to fine powder and an accurate amount equivalent to 0.285 g was dissolved in distilled water to prepare 1.0 × 10^−2^ mol L^−1^ standard solution. Serial dilutions were carried out to prepare different concentrations of LTZ within the range of 1.0 × 10^−10^–1.0 × 10^−2^ mol L^−1^. The fabricated sensors LTZ-PM, LTZ-PM-MgONPs and LTZ-PM-CuONPs were separately used to quantify the investigated drug.

### 2.11. Analysis of LTZ in Biosamples

To measure the concentration of LTZ in human plasma samples, approximately 3 mL of blood samples were collected from a forearm vein into vacuum heparinized tubes. The samples were withdrawn after 0.25–240 h of drug administration. The plasma samples were separated after centrifugation for 15 min at 1500 rpm and low temperature (less than 10 °C). Before and during the separation process, the samples were kept in an ice water bath. The obtained samples were analyzed using the fabricated modified sensors and LTZ concentrations in the plasma samples were calculated using regression equations.

### 2.12. Statistical Analysis

Statistical analyses were performed in triplicate measurements using Student’s *t*-test which was applied to compare the means between two groups at *p*-value < 0.05. The F test is used to evaluate the statistical variance significance [41].

## 3. Results and Discussion

### 3.1. Characterization of MgO and CuO Nanoparticles

**Figure 2:**
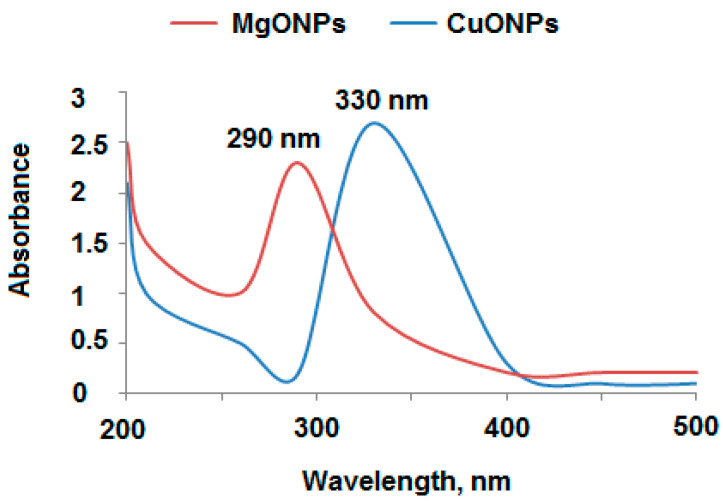
UV-Vis spectra of the synthesized MgONPs and CuONPs, the absorbance wavelength at 200–500 nm.

The prepared MgONPs and CuONPs were characterized using different spectroscopic methods. UV–Vis spectroscopy is the utmost useful and reliable technique suitable for confirming the primary characterization of size, shape and stability of the synthesized metal oxide nanostructures in their aqueous suspensions [42]. UV-Vis spectroscopy of MgONPs and CuONPs showed broad absorption peaks at 290 and 330 nm, respectively (Figure 2). The band gap was calculated using the formula Eg = hυ = hc/λ, where h is Planck’s constant, c is the velocity of light, and λ is the wavelength. The calculated band gab for each synthesized nanoparticles were 7.46 eV and 3.58 eV for MgONPs and CuONPs, respectively. The obtained results were in agreement with the standard band gaps of MgONPs (7.5 eV) and CuONPs (3.63 eV) previously reported in [43,44].

**Figure 3:**
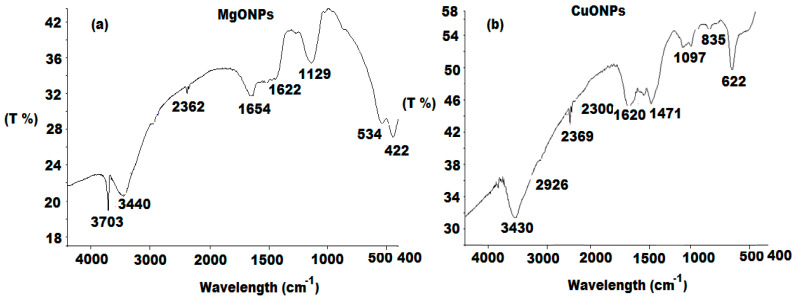
FT-IR spectra of synthesized (**a**) MgONPs and (**b**) CuONPs at a wavenumber range from 4000 to 400 cm^−1^.

FTIR analysis for MgONPs and CuONPs was performed in the range of 400–4000 cm^−1^. The absorption bands for MgONPs were 3703, 3440, 2362, 1654, 1622, 1129, 534 and 442 cm^−1^. The two bands observed at 3697 and 3646 cm^−1^ corresponded to the O–H bond stretching vibration. The weak band at 2362 cm^−1^ was assigned to be related to CO_2_ stretching vibration as a result of atmospheric carbon dioxide adsorption [45]. Two observed absorption bands around 1654 and 1622 cm^−1^ revealed the existence of an O-H stretching mode of water. A strong peak at 1129 cm^−1^ was attributed to S=O of sulfate. The noticed peak, which is slightly shifted from 534 to 442 cm^−1^ confirmed the formation of Mg-O stretching vibration (Figure 3a).

For CuONPs, two well defined bands at 3430 and 2926 cm^−1^ were observed and were related to both O-H and C-H stretching vibrations. The appearance of absorption band at 2360 cm^−1^ revealed the presence of CO_2_ stretching vibration. Additionally, the O–H stretching mode of water is confirmed by the presence of an absorption band at 1620 cm^−1^. In the range from 400–1000 cm^−1^, the observed bands around 835 and 622 cm^−1^ can be assigned to represent the formation of Cu–O (Figure 3b).

**Figure 4:**
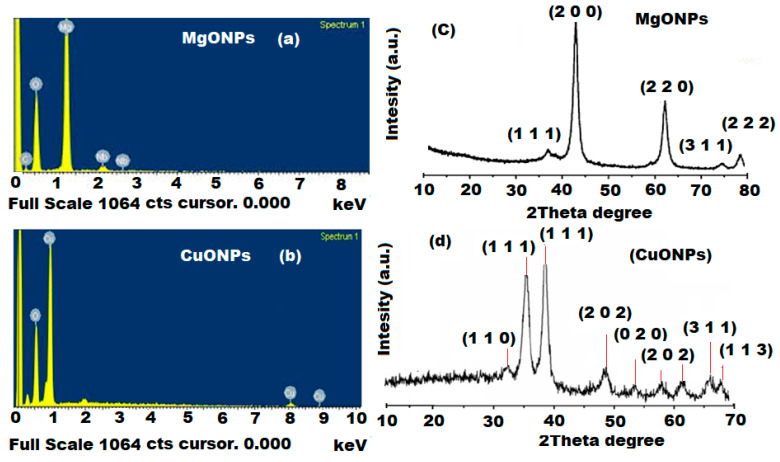
(**a**,**b**) EDX and (**c**,**d**) XRD spectra of MgONPs and CuONPs using XRD diffractometer with Cu-kα at (k = 1.5405 A°).

The presence of magnesium and copper elements in MgONPs and CuONPs was revealed by investigating their EDX profiles using SEM equipped with an EDX spectroscopy. The recorded profiles showed that the elemental composition percentage of Mg and Cu nanoparticles were 54.12% Mg and 45.88% O for MgONPs, while 72.48% Cu and 27.52% O in CuONPs (Figure 4a,b). The maximum intensity peaks were at 1.5 keV and 1.2 keV for Mg and Cu, respectively. This confirmed the high purity of the prepared nanoparticles and the reduction of magnesium and copper ion to zero valences. The outcomes were in agreement with the previously reported results in [46,47].

XRD is an analytical method for estimating and quantifying different crystalline forms in the tested samples. This analysis was carried out using XRD diffractometer with Cu-kα at (k = 1.5405 A°) and applied to determine and verify the crystal structure of MgONPs and CuONPs. XRD patterns of MgONPs displayed characteristic peaks at 2*θ* = 37.2°, 43.5°, 64.3°, 75.1° and 79.2° corresponding to MgO of (1 1 1), (2 0 0), (2 2 0), (3 1 1) and (2 2 2), respectively. These values can be indexed as a high hexagonal crystalline structure, and these results were similar to the JCPDS file of MgO (No. 36-1451). For CuONPs, different peaks were recorded at 2θ = 32.5°, 35.7°, 46.8° and 66.8° for CuO (1 1 1), (2 0 0), (2 0 2) and (1 1 3) plane orientation of CuO (JCPDS 80-1268). The broad XRD patterns revealed high particle crystalline and nanoscale dimensions. No other phases were observed and all diffraction peaks can be indexed as typical monoclinic structure (Figure 4c,d).

**Figure 5:**
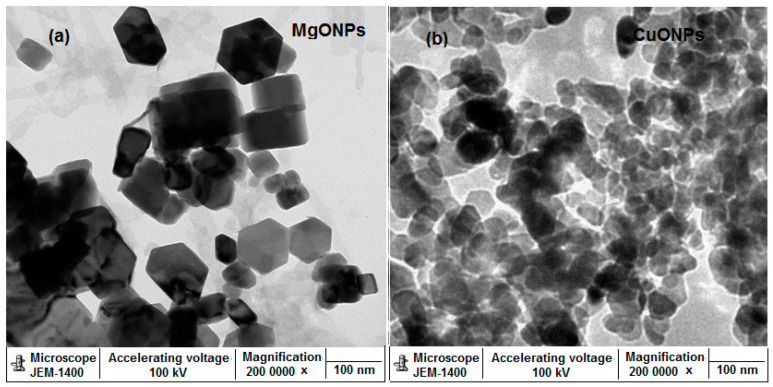
Transmission electron microscope (TEM) images of (**a**) MgONPs and (**b**) CuONPs at acceleration voltage 100 kV and magnification × 200,000.

**Figure 6:**
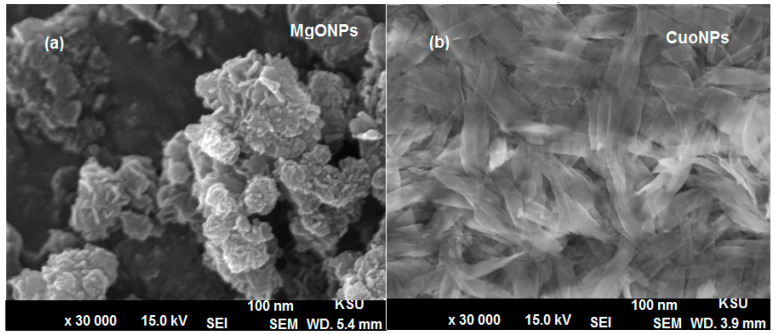
Scanning electron microscope (SEM) images of (**a**) MgONPs and (**b**) CuONPs at acceleration voltage 15.0 kV and magnification × 30,000.

Further microscopic investigations, including TEM and SEM, were carried out to study the surface size, shape and morphology of the prepared nanoparticles. The obtained images of MgONPs and CuONPs using TEM showed fairly uniform distributed particles with hexagonal and spherical shape for MgONPs and CuONPs, respectively. The recorded size of their particles was in the range from 60–100 nm for both MgONPs and CuONPs (Figure 5a,b). Moreover, the surface morphology of the synthesized metal oxide nanoparticles was studied under SEM using 30,000× magnification and the resulted images confirmed that they are highly aggregated crystals with particles size around 100 nm (Figure 6a,b).

### 3.2. The Nature of the Fabricated Sensors

LTZ interacts with PM to obtain a stable LTZ-PM complex soluble in THF. The fabrication of conventional and modified coated wire sensors was conducted by adding the active materials with (*o*-NPOE) as a solvent mediator in the presence of PVC. In the current study, *o*-NPOE acted as a fluidizer aiding homogenous dissolution of ion-pair and permitting its diffusion mobility inside the membrane. The elevated dielectric constant of *o*-NPOE (ε = 24) improves membrane selectivity towards the tested analyte by influencing the dissolution of ion pair within the active membrane and consequently increase its partition coefficient and gave suitable mechanical feature [48].

**Figure 7:**
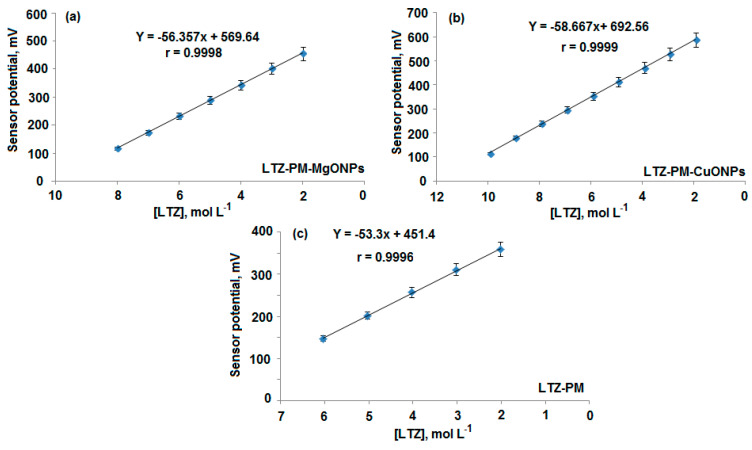
Calibration graphs for the determination of 1.0 × 10^−8^–1.0 × 10^−2^, 1.0 × 10^−10^–1.0 × 10^−2^ and 1.0 × 10^−6^–1.0 × 10^−2^ mol L^−1^ LTZ using the fabricated (**a**) modified LTZ-PM-MgONPs, (**b**) LTZ-CuONPs and (**c**) conventional LTZ-PM coated wire sensors, respectively.

Potentiometric response and critical characteristic performance of the LTZ-PM, LTZ-PM-MgONPs and LTZ-PM-CuONPs sensors were presented in Table 1. Outcomes showed that the above-mentioned sensors exhibited Nernstian responses with slopes of 53.3 ± 0.5, 56.4 ± 0.7 and 58.7 ± 0.5 mV over the drug concentration ranges of 10 × 10^−6^–1.0 × 10^−2^, 1.0 × 10^−8^–1.0 × 10^−2^ and 10 × 10^−10^–1.0 × 10^−2^ mol L^−1^ with correlation coefficients (0.9996, 0.9998, 0.9999) for conventional LTZ-PM, modified LTZ-PM-MgONPs and LTZ-PM-CuONPs, respectively (Figure 7a–c). The results showed that both modified metal oxide sensors displayed increased potentiometric response to a wide linear concentration range compared with the conventional one. Outcomes revealed high sensitivity of those sensors towards the determination of LTZ. This could be attributed to the coating nanoparticles layer with large surface area that enhanced the conductivity of the sensor surface. Furthermore, it was noticed that the use of CuONPs gave better results than MgONPs, which could be due to the elevated dielectric permittivity value of CuONPs (≈10^4^) over MgONPs (≈3.2–9.8) at room temperature [49,50].

The dynamic response (time of response) is known as the time between the instant at which the potential of the cell becomes equal to its steady-state value within 1 mV. This time should be taken under experimental conditions, including the constant stirring and precondition of the sensor in test sample prior to measuring the potential readings [51]. The dynamic response of each fabricated sensor was detected, and it was noticed that rapid dynamic responses at 75, 45 and 30 s for 20, 50 and 65 days were recorded for LTZ-PM, LTZ-PM-MgONPs and LTZ-PM-CuONPs, respectively. The results showed that the modified sensors enriched with metal oxide nanoparticles displayed fast and high stability compared with the conventional one. This could be due to the modification of sensors with nanomaterials, which possess new physicochemical features that are not present in the bulk material. These nanoparticles had greater surface to volume ratio improving interactions with targets in test solutions. Additionally, the extraordinary electrical properties, such as high charge transfer as well as the excellent electrical capabilities produced at interfaces of some nanostructured materials, are vital when nanomaterials are used as transductions in potentiometric sensors [52].

**Figure 8:**
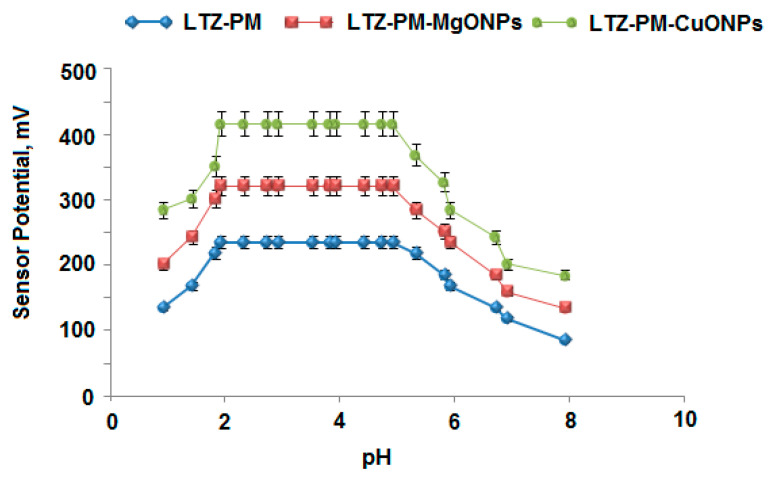
Effect of pH on the fabricated conventional LTZ-PM and modified metal oxide LTZ-PM-MgONPs and LTZ-PM-CuONPs coated wire sensors using 1.0 × 10^−4^ mol L^−1^ of LTZ solution.

The performance of the membrane sensors can greatly be influenced by hydrogen ions interference. Thus, the influence of pH on the potential of fabricated sensors was investigated to decide the safe pH range suitable for determining LTZ in its tested solutions. The outcomes demonstrated that both conventional and modified sensors were practically independent in the acidic pH range 2–5, and LTZ could simply be determined using the studied sensors within this range (Figure 8). It was observed that in acidic medium (below pH 2), the readings were slightly augmented due to the existence of H^+^ ions and the formation of protonated ion-pair that is poorly responsive to LTZ ions as well as the strong response to hydronium ions in the test solution. In the alkaline medium (pH value higher than 5), the readings were gradually decreased. The rise in OH^−^ ions caused a competition between LTZ and OH^−^ ions, and consequently decreased interaction between the tested drug ions and the sites of ion-pair on the sensor membrane. Therefore, the potential response of the fabricated sensors was reduced [53].

Selectivity of the fabricated LTZ-PM, LTZ-PM-MgONPs and LTZ-PM-CuONPs sensors towards the detection of LTZ using 1.0 × 10^−3^ mol L^−1^ was investigated. Separate solution method [40] was applied and various inorganic cations (Na^+^, K^+^, Ag^+^, Ni^2+^, Mg^2+^, Cu^2+^ and Zn^2+^), some sugars (glucose, lactose and starch) and amino acids (lysine, L. histidine, tryptophan, glycine, lysine, valine, and leucine) were tested. The presence of metal oxide nanoparticles with considerable surface area and physicochemical properties increased the conductivity of the constructed sensors, and hence increased selectivity towards the drug under investigation. This selectivity could be due to the free energy transfer of LTZ^+^ ions initiated between the membrane and the surrounding medium. The outcomes revealed the absence of any interference caused by sugars and amino acids. Additionally, the difference in inorganic cations ionic size, their mobility and permeability when compared with LTZ^+^ prevented the interference of these cations during the analysis. Thus, outstanding selectivity and suitable tolerance were achieved when LTZ-PM-MgONPs and LTZ-PM-CuONPs were used for determining LTZ (Table 2).

### 3.3. Quantification of Letrozole

The designed sensors were used to determine LTZ in its bulk powder. The direct calibration method was used and the obtained results were expressed as percentage recoveries. The outcomes of the analysis using the suggested sensors showed mean percentage recoveries of 98.9 ± 0.7, 99.6 ± 0.5% and 99.7 ± 0.3 for LTZ-PM, LTZ-PM-MgONPs and LTZ-PM-CuONPs, respectively (Table 3). These results displayed ultrasensitivity of the modified LTZ-PM-MgONPs and LTZ-PM-CuONPs sensors. The unique physicochemical characteristics of the used metal oxide nanoparticles enhanced the sensitivity and conductivity of the modified sensors towards the determination of the selected drug. Moreover, it was noticed that the CuONPs modified sensor exhibited an excellent detection towards the investigated LTZ due to the high dielectric constant of CuONPs over MgONPs.

### 3.4. Method Validation

The proposed analytical technique was ensured and validated according to ICH guidelines [54]. Wide linear concentration relationships were exhibited by the designed sensors over 1.0 × 10^−8^–1.0 × 10^−2^, 1.0 × 10^−10^–1.0 × 10^−2^ mol L^−1^, respectively, in comparison with 1.0 × 10^−6^–1.0 × 10^−2^ mol L^−1^ for the conventional coated wire type. The regression equations were estimated to be E_mV_ = (56.4 ± 0.7) log [LTZ] + 569.6 and E_mV_ = (58.7 ± 0.3) log [LTZ] + 692.6 for LTZ-PM-MgONPs and LTZ-PM-CuONPs, respectively. The conventional type LTZ-PM showed a potential response of E_mV_ = (53.3 ± 0.5) log [LTZ] + 451.4 with correlation coefficients 0.9998, 0.9999 and 0.9996 for the abovementioned sensors, respectively.

To detect the lower limit of detection (LOD), the potential readings of the designed sensors were recorded after the decrease in each sensor slope by 17.9 mV. The obtained LOD was 5.0 × 10^−7^, 5.9 × 10^−9^, and 5.6 × 10^−11^ mol L^−1^ for the three suggested sensors, respectively.

The accuracy of the developed potentiometric technique was investigated using nine authentic samples and the (mean ± SD) were estimated as 99.3 ± 0.4%, 99.6 ± 0.3% and 99.8 ± 0.3% for LTZ-PM, LTZ-PM-MgONPs and LTZ-PM-CuONPs, respectively. The intermediate precision of the proposed electrochemical procedure was evaluated using intra-day and inter-day assay and results were presented by estimating the relative standard deviation percentage (RSD %). The outcomes indicated that the RSD % for the fabricated LTZ-PM-MgONPs and LTZ-PM-CuONPs were 0.3% and 0.1%, 0.4% and 0.2% for intra-day and inter day, respectively. All results are less than the recommended value (2.0%) indicating high precise technique (Table 4).

The robustness of the described method was studied by changing the pH using acetate buffer pH 5 ± 0.5 and the (Mean ± SD) recoveries were recorded as 98.9 ± 0.6%, 99.2 ± 0.4% and 99.6 ± 0.1% for LTZ-PM, LTZ-MgONPs and LTZ-PM-CuONPs, respectively. An additional study was carried out to evaluate the raggedness of the proposed method by altering the pH meter model (Metrohm-744) The resulted (mean ± SD) recoveries were 99.2 ± 0.7%, 99.6 ± 0.5% and 99.8 ± 0.2% for the above-mentioned sensors. The outcomes confirmed a great agreement with those obtained by the described method.

### 3.5. Determination of LTZ in Tablets

To quantify the breast cancer medication LTZ in its pharmaceutical form (Femara^®^ 2.5 mg/tablet), the fabricated LTZ-PM, LTZ-PM-MgONPs and LTZ-PM-CuONPs sensors were used. The potential readings were measured vs. different concentrations of LTZ samples, and the recoveries percentage was estimated. The outcomes were 99.3 ± 0.4, 99.6 ± 0.3 and 99.9 ± 0.2 for the above-mentioned sensors, respectively (Table 5). It was observed that the modified sensor LTZ-PM-CuONPs displayed ultrasensitivity towards the determination of LTZ more than LTZ-PM-MgONPs. The enhancement of LTZ-PM-CuONPs conductivity over LTZ-PM-MgONPs one could be due to the higher dielectric constant of CuO over MgO.

The calculated (Mean ± SD) recoveries were assessed statistically using student’s *t*-test and F-test [41]. The results were compared with those achieved by the potentiometric method [37], which is established at the formation of PVC electrode using tetraphenylborate. The outcomes indicated excellent sensitivity of the proposed sensors towards the determination of LTZ in its dosage forms.

### 3.6. Quantification of LTZ in Biosamples

To confirm the suitability of the proposed modified metal oxide sensors for the detection of breast cancer medication letrozole, further investigations were carried out on 16 plasma samples of patients recommended to use letrozole as a breast cancer medication. The fabricated sensors were used to analyze the real samples withdrawn from women ranging from 25–55 years old. Certain increments (0.5 mol L^−1^ of LTZ) were added, and the potential concentration relationship was used to evaluate the tested drug in 3 replicates using the modified LYZ-PM-MgONPs and LTZ-PM-CuONPs. The results showed excellent efficiency for the quantification of LTZ with calculated RSD % (0.4–1.4%), (0.1–0.7%) and percentage recoveries (98.2–99.3%), (98.9–99.9%) for the sensors as represented in Table 6. Furthermore, a confirming study was carried out to compare the outcomes with other results obtained using the reported method [55]. The random analysis of plasma samples demonstrated that the modified sensors displayed ultrasenstivity for the quantification of LTZ when compared with the reported method.

## 4. Conclusions

The described electrochemical method was conducted by constructing two coated wire sensors modified with magnesium oxide and copper oxide nanoparticles. The suggested sensors were utilized for the determination of the oral non-steroidal medication for breast cancer (LTZ) in its authentic powder, commercial pharmaceuticals and biosamples. The measured potential readings of the modified sensors were compared with those of conventional LTZ-PM type. Outcomes of the modified sensors showed excellent and higher sensitivity over the conventional one due to the enhanced electro-conductivity. Additionally, the use of metal oxide nanoparticles as coated membrane modifiers promoted high selectivity in quantifying the selected drug with high selectivity with wide linear concentration range and low limit of detection. Thus, metal oxide enriched membrane sensors can successfully be applied for the analysis of LTZ in pharmaceutical industries, research laboratories and biosamples.

## Figures and Tables

**Table 1:** Performance characteristics of fabricated conventional coated wire LTZ-PM and modified LTZ-PM-MgONPs and LTZ-PM-CuONPs sensors.

Parameter	Conventional Coated Wire LTZ-PM Sensor	Modified LTZ-PM-MgONPs Sensor	Modified LTZ-PM-CuONPs Sensor
Slope (mV. Decade^−1^)	53.3 ± 0.5	56.4 ± 0.7	58.7 ± 0.5
Intercept	451.4	569.6	692.6
Regression equation	E_mV_ = (53.3 ± 0.5) log [LTZ] + 451.4	E_mV_ = (56.4 ± 0.7) log [LTZ] + 569.6	E_mV_ = (58.7 ± 0.5) log [LTZ] + 692.6
Correlation coefficient, r	0.9996	0.9998	0.9999
Linear range (mol L^−1^)	10 × 10^−6^–1.0 × 10^−2^	1.0 × 10^−8^–1.0 × 10^−2^	1.0 × 10^−10^–1.0 × 10^−2^
LOD	5.0 × 10^−7^	5.9 × 10^−9^	5.6 × 10^−11^
Response time/s	75	45	30
Working pH range	2–5	2–5	2–5
Lifetime/day	20	50	65
Temperature, °C	25	25	25
Accuracy (%)	99.3 ± 0.4	99.6 ± 0.3	99.8 ± 0.3

**Table 2:** Selectivity coefficient (K^Pot^_LTZ_^+^) of conventional coated wire LTZ-PM, modified LTZ-PM-MgONPs and LTZ-PM-CuONPs sensors by the separate solution method using 1.0 × 10^−3^ mol L^−1^ LTZ.

Interferences	Conventional Coated Wire LTZ-PM Sensor (K^pot^_LTZ_^+^)	Modified LTZ-PM-MgONPs Sensor (K^pot^_LTZ_^+^)	Modified LTZ-PM-CuONPs Sensor (K^pot^_LTZ_^+^)
Na^+^	5.4 × 10^−3^	4.8 × 10^−4^	9.2 × 10^−5^
K^+^	1.9 × 10^−3^	3.3 × 10^−4^	4.8 × 10^−4^
Ag^+^	3.1 × 10^−3^	1.5 × 10^−3^	8.4 × 10^−4^
Ni^2+^	5.6 × 10^−3^	4.2 × 10^−3^	2.2 × 10^−3^
Mg^2+^	6.8 × 10^−3^	7.9 × 10^−4^	8.7 × 10^−4^
Cu^2+^	6.6 × 10^−3^	1.4 × 10^−4^	2.3 × 10^−5^
Zn^2+^	4.9 × 10^−3^	4.9 × 10^−4^	6.3 × 10^−4^
Glucose	3.6 × 10^−3^	9.9 × 10^−4^	4.2 × 10^−4^
Lactose	3.9 × 10^−3^	6.7 × 10^−4^	5.6 × 10^−4^
Starch	4.8 × 10^−3^	2.3 × 10^−4^	2.1 × 10^−3^
Lysine	1.4 × 10^−3^	8.9 × 10^−3^	9.5 × 10^−4^
L-histidine	2.6 × 10^−3^	2.7 × 10^−4^	3.2 × 10^−5^
Tryptophan	5.5 × 10^−3^	5.8 × 10^−4^	5.4 × 10^−5^
Glycine	8.4 × 10^−3^	3.6 × 10^−3^	6.6 × 10^−4^
Valine	2.6 × 10^−3^	8.4 × 10^−3^	2.5 × 10^−4^
Leucine	2.5 × 10^−3^	3.5 × 10^−3^	2.9 × 10^−4^
Talc	5.2 × 10^−3^	2.2 × 10^−4^	9.1 × 10^−5^
SiO_2_	3.7 × 10^−3^	7.7 × 10^−3^	8.2 × 10^−4^
TiO_2_	7.8 × 10^−3^	7.8 × 10^−4^	7.8 × 10^−4^
Magnesium stearate	3.9 × 10^−3^	4.5 × 10^−3^	7.3 × 10^−5^
Microcrystalline cellulose	4.5 × 10^−3^	1.8 × 10^−4^	3.6 × 10^−4^

**Table 3:** The outcomes from the determination of LTZ in pure form using fabricated LTZ-PM, modified LTZ-PM-MgONPs and LTZ-PM-CuONPs coated wire sensors.

**Statistical analysis**	**Conventional LTZ-PM Coated Wire Sensor**		**Modified LTZ-PM MgONPs Sensor**		**Modified LTZ-PM CuONPs Sensor**	
	**Test Solution**	**% Recovery**	Test Solution *	**% Recovery**	Test Solution *	**% Recovery**
	6	98.5	8	99.8	10	100.0
	5.3	99.2	7	99.7	9	99.7
	5	99.8	6	100.0	8	99.9
	4	99.5	5	100.2	7	100.0
	3	98.0	4	99.8	6	99.7
	2	98.7	3	98.7	5	99.2
			2	99.0	4	99.8
					3	99.3
					2	99.5
Mean ± SD	98.9 ± 0.7		99.6 ± 0.5		99.7 ± 0.3	
n	6		7		9	
Variance	0.49		0.25		0.09	
RSD %	0.71		0.50		0.30	
SE **	0.29		0.18		0.12	

* Test solution using –log Conc. mol L^−1^. ** SE (standard error) = SD/$\sqrt{n}.$

**Table 4:** Intermediate precision assay of LTZ using modified LTZ-PM-MgONPs and LTZ-PM-CuONPs coated wire sensors.

**Statistical Analysis**	**Modified LTZ-PM-MgONPs Coated Wire Sensor**					
	**Intra-Day Assay**			**Inter-Day Assay**		
	Test Solution *	Found *	**% Recovery**	Test Solution *	Found *	**% Recovery**
	8.0	7.98	99.50	8.0	7.97	99.60
	6.0	6.00	100.00	6.0	5.99	99.80
	4.0	3.98	99.50	4.0	3.96	99.00
Mean ± SD	99.8 ± 0.3			99.5 ± 0.4		
n	3			3		
Variance	0.09			0.16		
RSD %	0.30			0.40		
SE **	0.17			0.23		
**Statistical Analysis**	**Modified LTZ-PM-CuONPs Sensor**					
	**Intra-day assay**			**Inter-day assay**		
	Test Solution *	Found *	**% Recovery**	Test solution *	Found *	**% Recovery**
	10	10.00	100.00	10	9.99	99.90
	8	7.99	99.90	8	8.00	100.00
	6	5.98	99.70	6	5.97	99.50
Mean ± SD	99.9 ± 0.1			99.8 ± 0.2		
n	3			3		
Variance	0.01			0.04		
RSD %	0.10			0.20		
SE **	0.06			0.12		

* Test solution using –log Conc. mol L^−1^. ** SE (standard error) = SD/$\sqrt{n}.$

**Table 5:** The outcomes from the determination of LTZ in Femara^®^ (2.5 mg Letrozole/tablet) using fabricated LTZ-PM, modified LTZ-PM-MgONPs and LTZ-PM-CuONPs coated wire sensors in comparison with previously reported method [37].

**Statistical Analysis**	**Conventional LTZ-PM Coated Wire Sensor**		**Modified LTZ-PM MgONPs Sensor**		**Modified LTZ-PM CuONPs Sensor**		**Reported Method [37]**
	Test Solution *	**% Recovery**	Test Solution *	**% Recovery**	Test Solution *	**% Recovery**	
	6	99.3	8	99.9	10	100.0	99.5 ± 0.4
	5.3	99.4	7	100.01	8	99.9	
	5	99.4	6	99.7	6	99.7	
	4	99.8	4	99.8	4	99.8	
	3	99.3	3	99.3	3	100.3	
	2	98.5	2	99.0	2	100.0	
Mean ± SD	99.3 ± 0.4		99.6 ± 0.3		99.9 ± 0.2		
n	6		6		6		
Variance	0.16		0.09		0.04		
RSD %	0.40		0.30		0.20		
SE **	0.16		0.12		0.08		
*t*-test	0.884 (2.228) ***		0.500 (2.228) ***		2.236 (2.228) ***		
F-test	1.00 (5.05) ***		1.78 (5.05) ***		4.00 (5.05) ***		

* Test solution and Found using –log Conc. mol L^−1^. ** SE (standard error) = SD/$\sqrt{n}$. *** The tabulated values of “Student’s *t*-test” and “F-test” at *p* < 0.05 [41].

**Table 6:** The outcomes from the determination of LTZ in biosamples using modified LTZ-PM-MgONPs and LTZ-PM-CuONPs coated wire sensors in comparison with a reported method [55].

Initial [LTZ], mol L^−1^	Added [LTZ] mol L^−1^	LTZ-PM-MgONPs	LTZ-PM-CuONPs	Reported Method [55]
		% Recovery ± %RSD	% Recovery ± %RSD	% Recovery ± %RSD
8.9	0.5	98.2 ± 0.8	99.3 ± 0.7	97.3 ± 0.6
6.8	0.5	98.4 ± 0.6	99.2 ± 0.1	96.8 ± 0.9
8.5	0.5	98.8 ± 0.5	99.5 ± 0.2	97.2 ± 1.2
6.6	0.5	98.2 ± 0.4	98.9 ± 0.6	96.8 ± 0.9
8.4	0.5	98.3 ± 0.5	99.4 ± 0.3	97.3 ± 0.7
6.3	0.5	98.7 ± 0.9	99.8 ± 0.2	98.2 ± 1.2
6.7	0.5	99.3 ± 0.7	99.5 ± 0.3	96.9 ± 1.4
8.7	0.5	99.2 ± 0.4	99.7 ± 0.9	97.6 ± 0.9
7.2	0.5	98.6 ± 1.2	99.9 ± 0.1	98.1 ± 0.4
8.3	0.5	98.6 ± 0.8	99.7 ± 0.3	96.7 ± 1.1
7.4	0.5	98.9 ± 1.2	99.2 ± 0.6	97.5 ± 0.6
8.1	0.5	98.2 ± 0.7	98.9 ± 0.7	98.1 ± 1.2
7.9	0.5	98.6 ± 0.4	99.4 ± 0.2	96.8 ± 0.9
8.8	0.5	99.3 ± 0.8	99.8 ± 0.4	97.8 ± 0.6
7.5	0.5	98.4 ± 1.4	99.6 ± 0.1	98.3 ± 0.4
8.4	0.5	99.3 ± 0.7	99.9 ± 0.3	96.9 ± 1.1

## Data Availability

All data are included within the text.
